# Suboptimal Vitamin D Status Is Associated with *Salmonella* Infection and Elevated *C*-Reactive Protein in Hospitalized Children with Acute Gastroenteritis: A Retrospective Cohort Study

**DOI:** 10.3390/nu18050827

**Published:** 2026-03-03

**Authors:** Hua-Hsi Hung, Hung-Chang Lee, Chun-Yan Yeung, Wai-Tao Chan, Szu-Wen Chang, Fang-Ju Sun, Chuen-Bin Jiang

**Affiliations:** 1 Department of Pediatric Gastroenterology, Hepatology and Nutrition, MacKay Children’s Hospital, Taipei 104217, Taiwan; shi03312003@gmail.com (H.-H.H.);; 2 Department of Medical Research, MacKay Memorial Hospital, Taipei 104217, Taiwan; 3 Department of Medicine, MacKay Medical University, New Taipei City 252005, Taiwan; 4 Department of Pediatric Gastroenterology, Hepatology and Nutrition, Hsinchu Municipal MacKay Children’s Hospital, Hsinchu City 300044, Taiwan; 5 Center for General Education, MacKay Junior College of Medicine, Nursing, and Management, Taipei 112021, Taiwan; 6 Institute of Biomedical Informatics, National Yang Ming Chiao Tung University, Taipei 112304, Taiwan

**Keywords:** vitamin D, *Salmonella*, *C*-reactive protein, acute gastroenteritis, pediatric patients, mucosal immunity

## Abstract

**Background:** Vitamin D contributes to intestinal barrier integrity and innate immune regulation, but its role in susceptibility to *Salmonella* infection and systemic inflammation in hospitalized pediatric patients with acute gastroenteritis (AGE) remains unclear. **Methods:** We retrospectively analyzed 70 pediatric patients hospitalized with AGE, examining the associations of admission 25(OH)D levels with culture-confirmed *Salmonella* infection and *C*-reactive protein (CRP). Multivariable logistic and linear regression models were adjusted for age and sex. Vitamin D status was classified as sufficient (≥30 ng/mL), suboptimal (<30 ng/mL), or deficient (<20 ng/mL). **Results:** The median age of the 70 included patients was 3.0 years (IQR: 1.5–6.0, range: 1.0–17 years), and 55.7% were male. Suboptimal vitamin D status was present in 64.3% of patients and was associated with higher median CRP levels (3.84 vs. 1.42 mg/dL, *p* = 0.025) and a greater prevalence of *Salmonella* infection (48.9% vs. 24.0%, *p* = 0.047). In multivariable analysis, suboptimal 25(OH)D levels independently marginally predicted an increased odds of *Salmonella* infection (adjusted odds ratio 3.11, 95% CI 1.00–9.67; *p* = 0.051). Serum 25(OH)D levels were inversely associated with natural log-transformed CRP (*p* = 0.026), with each 1 ng/mL increase corresponding to an estimated 5% reduction in CRP. Furthermore, vitamin D deficiency (<20 ng/mL) was associated with a 3.5-fold increase in CRP compared to levels ≥ 20 ng/mL (*p* = 0.012). **Conclusions:** Suboptimal vitamin D status (<30 ng/mL) may be associated with increased susceptibility to *Salmonella* gastroenteritis, while deficiency (<20 ng/mL) correlates with exacerbated inflammatory burden in hospitalized pediatric patients. These findings suggest a threshold-dependent effect of vitamin D on both mucosal defense and systemic inflammation. Prospective trials are warranted to evaluate the therapeutic potential of vitamin D supplementation in this population.

## 1. Introduction

Acute gastroenteritis (AGE) remains a leading cause of pediatric morbidity and mortality worldwide [1]. Although oral rehydration therapy has markedly reduced dehydration-related deaths, invasive bacterial gastroenteritis—particularly non-typhoidal *Salmonella* (NTS)—continues to pose substantial clinical challenges. Invasive NTS disease is associated with significant complications and mortality [2], and NTS remains an important cause of bacterial gastroenteritis globally [3]. In Taiwan, NTS is a leading bacterial etiology among pediatric patients hospitalized for AGE [4]. Compared with viral gastroenteritis, which is often mild and self-limiting, pediatric NTS infections more frequently present with high-grade fever, mucosanguineous stools, and marked systemic inflammation [5,6]. Furthermore, extra-intestinal dissemination and bacteremia can occur, particularly in young children [7]. Taken together, these features highlight the clinical need to identify modifiable host factors associated with *Salmonella* susceptibility and systemic inflammatory burden in hospitalized pediatric patients.

Beyond its classical role in bone metabolism, vitamin D has emerged as a key immunomodulator of the intestinal mucosal barrier and innate immune system [8,9]. The bioactive form, 1,25-dihydroxyvitamin D3, binds to the vitamin D receptor (VDR) and upregulates antimicrobial peptides (AMPs), including cathelicidin and β-defensins, which are relevant to host defense against enteric pathogens, including intracellular bacteria [10,11]. Vitamin D–VDR signaling has also been implicated in maintaining gut barrier integrity, including junctional function, which may mitigate bacterial translocation and downstream systemic inflammation [12,13].

Despite these mechanistic insights, clinical evidence linking vitamin D status to pathogen-specific susceptibility and inflammatory outcomes in pediatric populations remains fragmented and inconsistent. A recent systematic review and meta-analysis reported that vitamin D deficiency is significantly associated with an increased incidence of childhood diarrhea [14], and cross-sectional studies have similarly shown lower serum 25-hydroxyvitamin D [25(OH)D] levels in children with acute gastroenteritis compared with controls [15]. In addition, systematic reviews have highlighted vitamin D supplementation as a potential adjunctive approach in childhood infectious diarrhea, underscoring its translational relevance [16]. However, interventional data have been equivocal. In a pediatric randomized trial, vitamin D3 supplementation did not reduce diarrheal incidence [17], and a meta-analysis of randomized trials found no significant impact of vitamin D supplementation on serum CRP [18]. Importantly, most studies still assess “acute diarrhea” broadly without stratifying by specific pathogens. Consequently, it remains unclear whether admission 25(OH)D status is associated specifically with susceptibility to stool culture-confirmed *Salmonella* infection and whether it is also associated with systemic inflammatory burden (e.g., CRP) in hospitalized pediatric AGE. A further unresolved clinical question is the direction of this association—whether low 25(OH)D reflects a pre-existing vulnerability or decreases secondary to acute inflammation (reverse causality), thereby complicating causal interpretation [19,20].

To address this knowledge gap, we conducted a study focused on pediatric patients hospitalized with AGE to evaluate the association between admission serum 25(OH)D levels and (i) stool culture-confirmed *Salmonella* infection and (ii) systemic inflammatory burden as reflected by *C*-reactive protein (CRP). We hypothesized that suboptimal 25(OH)D levels at admission would be associated with higher odds of *Salmonella* positivity and higher CRP levels, potentially exhibiting a threshold-dependent pattern.

## 2. Materials and Methods

### 2.1. Study Design and Population

A retrospective cohort study was conducted at MacKay Children’s Hospital, a tertiary medical center in Taipei, Taiwan. We screened all pediatric patients (children and adolescents) aged 1 to <18 years admitted with AGE between 1 January and 31 December 2019. Age was defined as completed years at admission. AGE was clinically defined as the acute onset of diarrhea (≥3 loose or watery stools within a 24 h period), with or without associated symptoms such as vomiting or fever. The study period was specifically selected to encompass a full seasonal cycle while avoiding the epidemiologic shifts and potential confounding factors associated with the COVID-19 pandemic.

**Figure 1:**
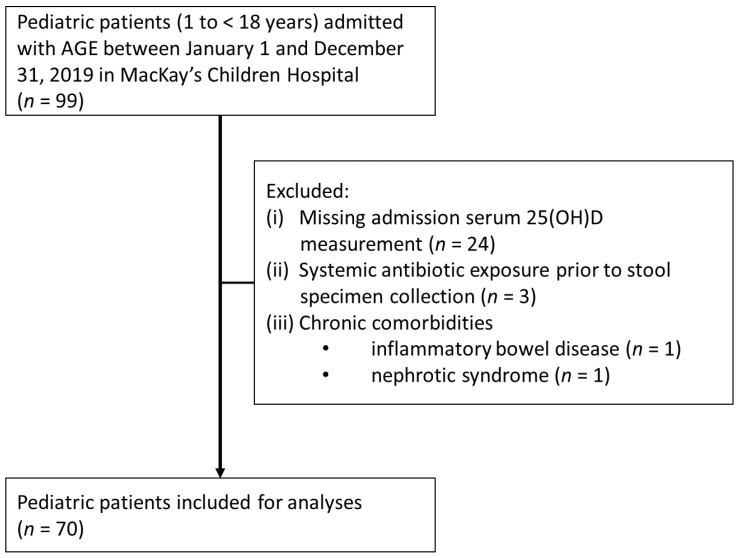
Flowchart of the study population. Chronic comorbidities included inflammatory bowel disease (*n *= 1) and nephrotic syndrome (*n *= 1). The primary reason for exclusion was missing admission serum 25(OH)D measurement (*n *= 24); additional exclusions were systemic antibiotic exposure prior to stool specimen collection (*n *= 3) and chronic comorbidities (*n *= 2). Abbreviations: AGE, acute gastroenteritis; 25(OH)D, 25-hydroxyvitamin D; CRP, *C*-reactive protein.

During the study period, the institutional inpatient protocol for pediatric AGE routinely included measurement of serum 25(OH)D and CRP at the time of admission, alongside the collection of stool specimens for bacterial culture for all hospitalized cases. Blood samples for 25(OH)D and CRP were collected at admission prior to intravenous fluid administration to minimize potential hemodilution. A total of 99 admissions met the AGE case definition. Patients were excluded for (i) missing admission serum 25(OH)D measurements (*n* = 24), (ii) systemic antibiotic exposure prior to stool specimen collection, or (iii) chronic comorbidities (inflammatory bowel disease or nephrotic syndrome). The final analytic cohort comprised 70 pediatric patients (Figure 1, Flowchart).

The study protocol was reviewed and approved by the Institutional Review Board of MacKay Memorial Hospital (Approval No. 26MMHIS026e).

### 2.2. Data Collection and Outcome Definitions

Clinical and laboratory data were extracted from electronic medical records using a standardized data abstraction form. Demographic variables included age and sex. Clinical indicators of disease severity included the presence of visible bloody stool and hospital length of stay (LOS).

The primary independent variable was the admission serum 25(OH)D level (measured by Abbott Laboratories, Abbott Park, IL, USA). Vitamin D status was categorized based on the Endocrine Society Clinical Practice Guidelines: sufficiency (≥30 ng/mL), insufficiency (20–29.9 ng/mL), and deficiency (<20 ng/mL) [21]. These cutoffs are primarily derived from bone-health criteria and are not specifically validated for infection-related outcomes; we used them for clinical interpretability and comparability with existing literature. For the primary analysis, “suboptimal vitamin D status” was defined as a serum 25(OH)D level < 30 ng/mL. The primary outcomes were: (i) *Salmonella* infection, defined as a positive stool culture for non-typhoidal *Salmonella* (NTS), and (ii) systemic inflammatory burden, assessed via admission CRP levels (measured by Beckman Coulter, Brea, CA, USA).

### 2.3. Statistical Analysis

Continuous variables were assessed for normality; those with non-normal distributions are presented as medians with interquartile ranges (IQRs) and compared using the Mann–Whitney U test. Categorical variables are reported as frequencies (%) and analyzed using the Chi-square test or Fisher’s exact test, as appropriate. Because admission CRP values exhibited a significant right-skewed distribution, they were natural log-transformed (lnCRP) for all parametric regression analyses.

To estimate the association between suboptimal vitamin D status and *Salmonella* infection, we employed multivariable logistic regression to calculate adjusted odds ratios (aORs) with 95% confidence intervals (CIs). Multivariable linear regression was used to evaluate the relationship between 25(OH)D and lnCRP. Coefficients from the linear models were exponentiated to provide geometric mean ratios (GMRs). All multivariable models were adjusted a priori for age and sex to account for potential confounding. Severity indicators (e.g., bloody stool, LOS) were examined in univariate analyses but excluded from the primary adjusted models to avoid over-adjustment bias from potential mediators. Statistical significance was defined as a two-sided *p*-value < 0.05. All analyses were performed using SPSS (version 26; IBM Corp., Armonk, NY, USA).

### 2.4. Sample Size and Power Considerations

Because this was a retrospective cohort study, the sample size was determined by the number of eligible admissions with available admission serum 25(OH)D, CRP, and stool culture data (*N* = 70). Power calculations were performed using G*Power (version 3.1.9.4; Heinrich Heine University Düsseldorf, Düsseldorf, Germany; two-sided α = 0.05). For the inflammatory endpoint, the continuous 25(OH)D variable in the adjusted model yielded an achieved power of approximately 0.67 (partial *R*^2^ = 0.073, *f*^2^ = 0.085). For the *Salmonella* endpoint, power was estimated using a two-proportion framework based on the observed event rate and allocation ratio, yielding an achieved power of approximately 0.59; this limited precision is acknowledged as a limitation, and larger prospective studies are needed. Covariates were restricted to age and sex to minimize overfitting given the number of *Salmonella* events (*n *= 28).

## 3. Results

### 3.1. Demographic Characteristics and Prevalence of Vitamin D Status

A total of 70 pediatric patients hospitalized with acute gastroenteritis (AGE) were included in the study. A comparison between included and excluded admissions is provided in Supplementary Table S1. Compared with excluded admissions, the included group more frequently presented with visible bloody stool (62.9% vs. 31.0%, *p* = 0.004) and had a longer length of stay [5.00 (4.00–6.00) vs. 4.00 (3.00–5.50) days, *p* = 0.020], with a trend toward higher admission CRP [3.13 (0.95–7.65) vs. 1.11 (0.09–6.62) mg/dL, *p* = 0.057]. The prevalence of suboptimal vitamin D status [25(OH)D < 30 ng/mL] was 64.3% (*n *= 45), while 35.7% (*n *= 25) of the cohort exhibited sufficient levels [25(OH)D ≥ 30 ng/mL] (Table 1). Baseline demographic characteristics, including age and sex, were comparable between the suboptimal and sufficient groups (median age: 3.00 vs. 2.00 years, *p* = 0.259; male: 51.1% vs. 64.0%, *p* = 0.298). Furthermore, no significant seasonal variation in serum 25(OH)D levels was observed (*p* = 0.985; Table S2).

### 3.2. Clinical Outcomes Stratified by Vitamin D Status

Pediatric patients with suboptimal vitamin D status presented with a significantly higher systemic inflammatory burden and a greater prevalence of *Salmonella* infection compared to those with sufficient levels (Table 1). Specifically, the median admission CRP was markedly elevated in the suboptimal group (3.84 vs. 1.42 mg/dL, *p* = 0.025). The prevalence of culture-confirmed *Salmonella* infection was also significantly higher among pediatric patients with suboptimal status (48.9% vs. 24.0%, *p* = 0.047). Although visible bloody stool was more frequently documented in the suboptimal group (71.1% vs. 48.0%), this difference did not reach statistical significance (*p* = 0.073).

### 3.3. Association Between Vitamin D Status and Salmonella Infection

In the univariate logistic regression analysis, suboptimal 25(OH)D (<30 ng/mL) was associated with significantly higher odds of *Salmonella* infection (OR 3.03, 95% CI 1.02–8.99; *p* = 0.046) (Table 2). After adjusting for age and sex, the multivariable model yielded a similar point estimate, indicating a more than three-fold increase in the odds of *Salmonella* positivity (aOR 3.11, 95% CI 1.00–9.67), although the association was marginally above the conventional significance threshold (*p* = 0.051).

### 3.4. Impact of Vitamin D Status on Systemic Inflammation

Univariate associations between clinical characteristics and admission lnCRP are presented in Table S3. Multivariable linear regression models demonstrated a consistent inverse relationship between vitamin D status and admission lnCRP (Table 3). In the continuous model adjusted for age and sex, serum 25(OH)D was inversely associated with lnCRP (*B* = −0.050, exp(*B*) = 0.95, 95% CI 0.91–0.99; *p* = 0.026). This indicates that for every 1 ng/mL increase in 25(OH)D, there was a corresponding 5% reduction in the geometric mean of CRP.

**Figure 2:**
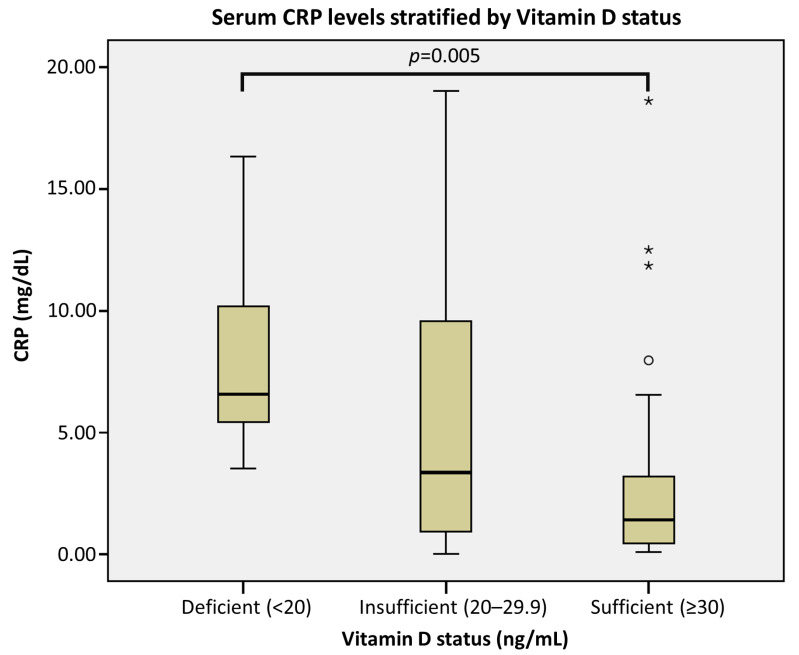
Serum *C*-reactive protein (CRP) levels stratified by Vitamin D status. Box plot showing admission CRP across three groups: deficient (<20 ng/mL), insufficient (20–29.9 ng/mL), and sufficient (≥30 ng/mL). Circles (o) represents mild outliers (values between 1.5 and 3 interquartile ranges [IQR] from the box edge), and asterisks (*) represents extreme outliers (values more than 3 IQR from the box edge). Group comparisons were performed using one-way ANOVA on natural log-transformed CRP (lnCRP) with post hoc testing; the *p* value displayed indicates the pairwise comparison between the deficient and sufficient groups.

Categorical analyses further revealed a distinct threshold-like pattern for systemic inflammation. While the association for the suboptimal cutoff (<30 ng/mL) did not reach statistical significance in the adjusted model (*p* = 0.092), vitamin D deficiency (<20 ng/mL) was associated with substantially higher CRP levels compared with the non-deficient group (≥20 ng/mL) (exp(*B*) = 3.49, 95% CI 1.33–9.13; *p* = 0.012). Consistently, CRP values were markedly elevated in the deficient group in the boxplot stratified by vitamin D status (Figure 2).

Taken together, these data suggest that while a broader suboptimal status (<30 ng/mL) is linked to *Salmonella* susceptibility, a more severe deficiency (<20 ng/mL) is required to drive a pronounced systemic inflammatory response.

## 4. Discussion

In this retrospective investigation of pediatric patients hospitalized with AGE, we identified two salient patterns linking vitamin D status to clinical outcomes. First, suboptimal vitamin D levels (<30 ng/mL) were associated with a threefold increase in the odds of culture-confirmed *Salmonella* infection. Second, a distinct threshold effect was observed for systemic inflammation, where a significant surge in CRP levels was confined primarily to pediatric patients with vitamin D deficiency (<20 ng/mL). These findings suggest that while a broader “suboptimal” status may compromise mucosal defense against invasive pathogens, a more profound “deficiency” is required to dysregulate systemic inflammatory control.

### 4.1. Suboptimal Vitamin D Status and Salmonella Susceptibility

Non-typhoidal *Salmonella* remains a substantial burden in pediatric AGE, characterized by more severe inflammatory phenotypes compared to viral etiologies [5,6]. In addition, invasive NTS disease has been associated with significant complications and mortality, and pediatric series have documented extra-intestinal dissemination and bacteremia, highlighting the importance of identifying host factors related to NTS susceptibility in hospitalized pediatric patients [2,7]. Our finding of an independent association between lower 25(OH)D and *Salmonella* positivity aligns with emerging evidence from invasive enteritis cohorts [22]. Whereas prior clinical studies largely evaluated childhood diarrhea without pathogen stratification [14,15], our culture-confirmed analysis provides pathogen-specific insight into host–pathogen interactions.

Biologically, this association is plausible. Vitamin D receptor (VDR) signaling is a critical regulator of mucosal innate immunity. Upon activation, VDR induces the expression of antimicrobial peptides (AMPs), such as cathelicidin (LL-37) and β-defensins, which are essential for the direct neutralization of intracellular pathogens [10,11]. Furthermore, given the facultative intracellular nature of *Salmonella*, vitamin D-mediated autophagy represents another vital defense mechanism. Experimental models have demonstrated that VDR signaling enhances autophagic clearance of *Salmonella* in intestinal epithelial cells [23,24]. We postulate that suboptimal 25(OH)D levels (<30 ng/mL) may impair these first-line mucosal defenses, thereby increasing susceptibility to *Salmonella* colonization and invasion, even before systemic inflammation becomes dysregulated.

### 4.2. Threshold-Dependent Regulation of Systemic Inflammation

A novel and clinically relevant finding of our study is the distinct threshold pattern observed for systemic inflammation. While susceptibility to *Salmonella* infection was observed at the suboptimal level (<30 ng/mL), a significant surge in CRP levels was confined primarily to pediatric patients with vitamin D deficiency (<20 ng/mL). This non-linear relationship suggests that while mucosal immunologic defense may be compromised at suboptimal levels, the “brake” on systemic inflammatory cascades remains functional until vitamin D stores are profoundly depleted.

This clinical observation mirrors emerging genetic evidence regarding the causal directionality between vitamin D and inflammation. A recent large-scale bidirectional Mendelian randomization (MR) study by Zhou and Hyppönen (2023) analyzed data from over 290,000 participants and found that genetically predicted lower 25(OH)D was causally associated with higher CRP levels [20]. Crucially, they identified an L-shaped association where the inflammatory effect was most pronounced in the deficiency range and plateaued at approximately 50 nmol/L (~20 ng/mL). Our finding of a steep rise in CRP specifically in the <20 ng/mL group [exp(B) = 3.49] provides clinical corroboration of this genetic “tipping point,” reinforcing the hypothesis that vitamin D deficiency acts as a driver of systemic inflammation rather than merely a consequence.

Mechanistically, this threshold likely reflects the requirement for sufficient intracellular 1,25(OH)_2_D_3_ to maintain the physical integrity of the intestinal barrier. Assa et al. (2014) demonstrated in murine models that severe vitamin D deficiency is required to disrupt tight junction proteins (e.g., claudins and occludin) and increase epithelial permeability to luminal antigens [25]. Similarly, Wu et al. (2010) reported that VDR signaling negatively regulates bacterial-stimulated NF-*κ*B activity in the intestine, preventing exaggerated pro-inflammatory cytokine responses [26]. It is plausible that in our cohort, pediatric patients with levels between 20–30 ng/mL retained sufficient VDR signaling to suppress systemic NF-*κ*B activation and maintain barrier function, whereas those with levels < 20 ng/mL experienced a dual failure of pathogen clearance and inflammatory regulation.

A critical challenge in interpreting these findings is the potential for reverse causality, as 25(OH)D behaves as a negative acute-phase reactant and may decline during acute inflammation [19]. While our cross-sectional design cannot fully disentangle temporal sequences, recent genetic evidence supports a causal direction from low vitamin D to increased inflammation. A large-scale bidirectional Mendelian randomization (MR) study demonstrated that genetically predicted lower 25(OH)D is causally associated with higher CRP, whereas genetically elevated CRP does not significantly lower 25(OH)D [20]. Notably, this MR analysis identified a non-linear effect that plateaued around 20 ng/mL, which strikingly mirrors the cutoff where we observed the most profound inflammatory signal. This concordance is consistent with the hypothesis that the low vitamin D status observed in our high-CRP patients may be a contributing factor, rather than solely a consequence of, the inflammatory state; however, because measurements were obtained at admission, temporality cannot be established and reverse causality cannot be excluded.

### 4.3. Strengths and Limitations

This study possesses several notable strengths. First, by focusing on a clinically relevant cohort of hospitalized pediatric patients with acute gastroenteritis (AGE) and utilizing stool culture-confirmed *Salmonella*, we provide pathogen-specific inferences rather than relying on broad, syndromic “acute diarrhea” classifications [14,15]. Second, the study period spanned a complete calendar year during the pre-COVID-19 era, effectively mitigating potential seasonal variations in 25(OH)D and avoiding epidemiologic shifts that could influence pathogen distribution and hospital admission thresholds. Third, key laboratory parameters (25(OH)D and CRP) were systematically obtained at admission as part of a standardized inpatient workflow, ensuring a consistent baseline assessment of both pathogen positivity and systemic inflammatory burden. Finally, we prespecified a parsimonious multivariable modeling strategy to reduce the risk of statistical overfitting while rigorously accounting for major demographic confounders.

However, several limitations warrant careful consideration. First, the retrospective, cross-sectional nature of the exposure and outcome assessments at admission precludes the establishment of temporality and limits causal inference. Because circulating 25(OH)D may decrease secondary to acute inflammation (acting as a negative acute-phase reactant), reverse causality remains a plausible alternative explanation [19,20]. Second, the relatively small sample size (*N *= 70) limits statistical power, particularly for the *Salmonella* endpoint where the adjusted association was borderline and the confidence interval was wide (aOR 3.11, 95% CI 1.00–9.67), indicating some imprecision and reduced ability to detect modest between-group differences.

Third, our multivariable models were adjusted strictly for age and sex; therefore, residual confounding cannot be completely ruled out. We lacked detailed documentation on several pre-admission factors—such as precise nutritional status (BMI), prior vitamin D supplementation, sunlight exposure, and the initial degree of dehydration—that could influence both 25(OH)D levels and clinical outcomes. Although we rigorously excluded patients who received antibiotics prior to stool culture collection, confounding related to the broader pre-admission clinical course remains possible.

Fourth, a substantial proportion of eligible admissions were excluded due to missing admission 25(OH)D data, which introduces the potential for selection bias. Our analytic cohort tended to present with a longer hospital length of stay, higher CRP levels, and a greater frequency of visible bloody stools, suggesting an enrichment for more severe hospitalized cases. Moreover, this hospital-based sampling strategy may introduce Berkson’s bias, enriching the cohort for specific combinations of exposure and disease severity. Consequently, the extrapolation of these findings to milder, outpatient cases of AGE should be approached with caution.

Finally, as a single-center study conducted in Taiwan, the generalizability of these findings to other ethnic groups or geographic regions with distinct *Salmonella* serotype prevalence requires further validation. Future prospective cohort studies integrating systematic measurements of key confounders and, ultimately, randomized interventional trials are necessary to clarify causality and establish clinical utility.

## 5. Conclusions

In conclusion, this study highlights a dual role for vitamin D in pediatric acute gastroenteritis: suboptimal status (<30 ng/mL) may be linked to increased susceptibility to *Salmonella* infection, while deficiency (<20 ng/mL) is associated with heightened systemic inflammation. These findings suggest that maintaining sufficient vitamin D levels may be crucial for both mucosal defense and inflammatory regulation. Future prospective trials are warranted to determine whether vitamin D supplementation can serve as an adjunctive strategy to reduce infection risk and mitigate inflammatory severity in hospitalized pediatric patients.

## Figures and Tables

**Table 1:** Demographic and Clinical Characteristics Stratified by Vitamin D Status.

Variable	Total (*N* = 70)	25(OH)D ≥ 30 ng/mL (*n* = 25)	25(OH)D < 30 ng/mL (*n* = 45)	*p*
Age (years), Median (IQR)	2.00 (1.75–5.00)	2.00 (1.50–3.00)	3.00 (1.50–6.00)	0.259
Male, n (%)	39 (55.7%)	16 (64.0%)	23 (51.1%)	0.298
Length of stay (LOS), Median (IQR)	5.00 (4.00–6.00)	5.00 (4.00–5.50)	5.00 (4.00–6.00)	0.372
CRP (mg/dL), Median (IQR)	3.13 (0.95–7.65)	1.42 (0.43–4.05)	3.84 (1.05–9.93)	0.025 *
Visible Bloody Stool, n (%)	44 (62.9%)	12 (48.0%)	32 (71.1%)	0.073
Culture-confirmed *Salmonella*, n (%)	28 (40.0%)	6 (24.0%)	22 (48.9%)	0.047 *

Data are presented as median (interquartile range, IQR) for continuous variables and number (percentage, %) for categorical variables. *p*-values represent the statistical comparison between the 25(OH)D ≥ 30 ng/mL and 25(OH)D < 30 ng/mL groups. Differences between groups were assessed using the Mann–Whitney U test for continuous variables and the Chi-square (χ^2^) test or Fisher’s exact test for categorical variables. * *p* < 0.05 indicates statistical significance. Note: The full age range of the included cohort was 1–17 years. Abbreviations: 25(OH)D, serum 25-hydroxyvitamin D; CRP, *C*-reactive protein; LOS, hospital length of stay; IQR, interquartile range.

**Table 2:** Univariate and Multivariable Logistic Regression Analyses for Predictors of Stool Culture–confirmed *Salmonella* Infection.

	Univariate Analysis		Multivariable Analysis †	
Variable	OR (95% CI)	*p*	aOR (95% CI)	*p*
Suboptimal 25(OH)D (<30 ng/mL)	3.03 (1.02–8.99)	0.046 *	3.11 (1.00–9.67)	0.051
Age (years, per 1-year increase)	0.96 (0.84–1.11)	0.590	0.94 (0.81–1.09)	0.431
Sex, male	0.42 (0.16–1.11)	0.080	0.46 (0.17–1.27)	0.136
Visible bloody stool, yes ‡	2.48 (0.87–7.08)	0.090	—	—
Hospital Length of stay (days, per 1-day increase) ‡	1.2 (0.98–1.46)	0.081	—	—

Data are presented as odds ratios (ORs) for univariate analysis and adjusted odds ratios (aORs) for multivariable analysis, with 95% confidence intervals (CIs). † The primary multivariable model included suboptimal vitamin D status (<30 ng/mL) and was adjusted a priori for age and sex. ‡ Clinical severity indicators (visible bloody stool and hospital length of stay) were evaluated in univariate analyses but excluded from the multivariable model to avoid over-adjustment bias, as they represent potential downstream mediators of infection. —Indicates variables not included in the model. Reference categories: 25(OH)D ≥ 30 ng/mL; female sex; no visible bloody stool. * *p* < 0.05 indicates statistical significance. Abbreviations: 25(OH)D, serum 25-hydroxyvitamin D; OR, odds ratio; aOR, adjusted odds ratio; CI, confidence interval.

**Table 3:** Linear Regression Models Evaluating the Association Between Serum 25(OH)D Levels and Admission CRP (lnCRP).

		Model 1	Model 2	Model 3	Model 4
Analysis Type		Univariate	Multivariable †	Multivariable †	Multivariable †
25(OH)D specification		Continuous	Continuous	Categorical (<30 vs. ≥30)	Categorical (<20 vs. ≥20)
Variables					
25(OH)D	*B* (95% CI)	−0.060 (−0.101 to −0.018)	−0.050 (−0.093 to −0.006)	0.647 (−0.109 to 1.404)	1.249 (0.287 to 2.212)
	exp(*B*) (95% CI)	0.94 (0.90–0.98)	0.95 (0.91–0.99)	1.91 (0.90–4.07)	3.49 (1.33–9.13)
	*p*	0.006 **	0.026 *	0.092	0.012 *
Age (years)	*B* (95% CI)	—	0.081 (−0.022 to 0.184)	0.097 (−0.005 to 0.200)	0.092 (−0.007 to 0.191)
	*p*	—	0.119	0.063	0.069
Sex, male	*B* (95% CI)	—	−0.127 (−0.837 to 0.583)	−0.155 (−0.876 to 0.566)	−0.148 (−0.847 to 0.551)
	*p*	—	0.722	0.669	0.674

The dependent variable was natural log-transformed *C*-reactive protein (lnCRP). Data are presented as unstandardized regression coefficients (*B*) and exponentiated coefficients [exp(*B*)], with 95% confidence intervals (CIs). † Models 2–4 were adjusted a priori for age and sex. Interpretation of exp(*B*): Reported as the geometric mean ratio (GMR) of CRP. For continuous models (Models 1–2): GMR represents the multiplicative change in CRP levels for every 1 ng/mL increase in 25(OH)D. For categorical models (Models 3–4): GMR represents the ratio of geometric mean CRP levels in the specified group relative to the reference group (Reference: ≥30 ng/mL for Model 3; ≥20 ng/mL for Model 4). * *p* < 0.05, ** *p* < 0.01 indicates statistical significance. Abbreviations: 25(OH)D, serum 25-hydroxyvitamin D; CRP, *C*-reactive protein.

## Data Availability

The data presented in this study are available upon reasonable request from the corresponding author. The data are not publicly available due to privacy reasons.

## Supplementary Materials

The following supporting information can be downloaded at: https://www.mdpi.com/article/10.3390/nu18050827/s1, Table S1: Comparison of Demographic and Clinical Characteristics Between Included and Excluded Patients; Table S2: Seasonal variation of serum 25(OH)D levels; Table S3: Univariate linear regression analyses for admission lnCRP.

## Abbreviations

The following abbreviations are used in this manuscript: AGE, acute gastroenteritis; 25(OH)D, serum 25-hydroxyvitamin D; CRP, *C*-reactive protein; lnCRP, natural log-transformed CRP; NTS, non-typhoidal *Salmonella*; LOS, length of stay; IQR, interquartile range; OR, odds ratio; aOR, adjusted odds ratio; CI, confidence interval; GMR, geometric mean ratio; VDR, vitamin D receptor; AMPs, antimicrobial peptides; MR, Mendelian randomization; NF-κB, nuclear factor kappa B; ANOVA, analysis of variance; SPSS, Statistical Package for the Social Sciences.
