# Supplementary material for: Suboptimal Vitamin D Status Is Associated with Salmonella Infection and Elevated C-Reactive Protein in Hospitalized Children with Acute Gastroenteritis: A Retrospective Cohort Study
Source: Nutrients. 2026 Mar 3;18(5):827. doi: 10.3390/nu18050827 (PMC12986610; doi:10.3390/nu18050827)
Supplement: Supplementary file 1 [file nutrients-18-00827-s001.zip › nutrients-4150229-supplementary.pdf]

Supplementary Materials

Table S1. Comparison of Demographic and Clinical Characteristics Between Included and Excluded Patients.

Variable	Included (n = 70)	Excluded (n = 29)	*p*
Age (years), Median (IQR)	2.00 (1.75 – 5.00)	2.00 (1.00 – 5.00)	0.201
Male, n (%)	39 (55.7%)	18 (62.1%)	0.560
Visible bloody stool, n(%)	44(62.9%)	9(31.0%)	0.004*
LOS (days), Median (IQR)	5.00 (4.00 – 6.00)	4.00 (3.00 – 5.50)	0.020*
CRP (mg/dL), Median (IQR)	3.13 (0.95 – 7.65)	1.11 (0.09 – 6.62)	0.057
Culture-confirmed *Salmonella*, n (%)	28/70 (40.0%)	5/26 (19.2%) †	0.089 ‡

Data are presented as median (interquartile range, IQR) for continuous variables and number (percentage, %) for categorical variables. *p*-values represent comparisons between the Included and Excluded groups. Differences were assessed using the Mann–Whitney U test for continuous variables and the Chi-square (χ^2) test or Fisher's exact test for categorical variables. * $p < 0.05$ indicates statistical significance. † Three patients in the Excluded group received antibiotics prior to stool culture collection and were therefore excluded from *Salmonella* analysis (n = 26 for this comparison). ‡ Fisher's exact test (2-sided). Abbreviations: CRP, C-reactive protein; IQR, interquartile range; LOS, hospital length of stay.

Table S2. Seasonal Variation of Serum 25(OH)D Levels

Season	N	25(OH)D Level (ng/mL)(Mean ± SD)	*p*
Spring (Mar – May)	19	27.98 ± 7.60	
Summer (Jun – Aug)	36	28.07 ± 8.89	
Autumn (Sep – Nov)	12	27.74 ± 8.23	
Winter (Dec – Feb)	3	29.87 ± 12.52	
Total	70	28.08 ± 8.35	0.985

Notes: Values are presented as mean ± SD because comparisons across seasons were performed using one-way analysis of variance (ANOVA). Two-tailed *p* values are shown.

Table S3. Univariate linear regression analyses for admission CRP (lnCRP)

Variables	*B* (95% CI)	*p*
25(OH)D (per 1 ng/mL increase)	−0.060 (−0.101, −0.018)	0.006**
Age (years)	0.110 (0.009, 0.212)	0.033*
Sex (male)	−0.175 (−0.917, 0.567)	0.639
Length of stay (days)	0.098 (−0.034, 0.230)	0.144
Visible bloody stool (yes)	0.401 (−0.357, 1.158)	0.295

Note: The dependent variable was natural log-transformed CRP (lnCRP). Values represent the unstandardized linear regression coefficient (*B*) and 95% confidence intervals (CI). * $p < 0.05$; ** $p < 0.01$ indicates statistical significance. Abbreviations: CRP, C-reactive protein; 25(OH)D, serum 25-hydroxyvitamin D
